# An Unusual Case of Acquired Dilated Cardiomyopathy Due to Long-Standing Patent Ductus Arteriosus

**DOI:** 10.7759/cureus.101436

**Published:** 2026-01-13

**Authors:** Eleni Giannopoulou, Damianos Tsilivarakis, Maria Lekaditi, Stavroula Kosmopoulou

**Affiliations:** 1 Department of Cardiology, General Hospital of Kalamata, Kalamata, GRC; 2 Department of Pediatrics, General Hospital of Kalamata, Kalamata, GRC

**Keywords:** adult congenital heart disease (achd), dilated cardiomyopathy (dcm), heart failure with reduced ejection fraction, left-to-right shunt, patent ductus arteriosus (pda)

## Abstract

Patent ductus arteriosus (PDA) is a common congenital cardiac condition in neonates. Its frequency is substantially higher in preterm infants, and it is frequently associated with other congenital heart defects. Its persistence into adulthood has become increasingly uncommon due to advances in neonatal screening, echocardiographic diagnosis, and timely therapeutic intervention. The hemodynamic burden imposed by PDA depends on the magnitude of the left-to-right shunt and may result in both short- and long-term complications. While small shunts may remain asymptomatic throughout life, moderate and large shunts can cause pulmonary congestion, cardiac volume overload, organ hypoperfusion, and even death, early in life. When left untreated, particularly in small-to-moderate defects that may not initially meet the criteria for intervention, chronic shunting may progressively result in pulmonary hypertension and heart failure. In adults, heart failure related to PDA is usually associated with preserved or moderately reduced left ventricular ejection fraction (LVEF), whereas severe systolic dysfunction and acquired dilated cardiomyopathy are exceptionally rare. Here, we present the case of a 70-year-old male who was admitted with progressive dyspnea, orthopnea, dry cough, and bilateral lower extremity edema over one month. His medical history included PDA diagnosed in childhood, without intervention or regular follow-up. On admission, physical examination revealed signs of acute heart failure, including tachypnea, jugular venous distension, pulmonary congestion, and marked peripheral edema. Cardiac auscultation demonstrated a prominent second heart sound and systolic murmurs consistent with mitral and tricuspid regurgitation, and a characteristic continuous, “machinery” murmur suggestive of PDA. Electrocardiography showed sinus rhythm with frequent atrial and ventricular premature beats, poor R-wave progression in the precordial leads, and nonspecific ST-T abnormalities in the limb leads. Chest radiography demonstrated cardiomegaly, pulmonary vascular congestion, alveolar edema, and bilateral pleural effusions. Laboratory evaluation revealed elevated natriuretic peptide levels and mildly increased high-sensitivity cardiac troponin I, consistent with the setting of acute heart failure. Transthoracic echocardiography revealed dilation of all four chambers and severe biventricular systolic dysfunction, with an LVEF of 25%, mild mitral and tricuspid insufficiency, and increased systolic pressure in the pulmonary artery. A moderate left-to-right shunt was found. Evaluation by coronary angiography ruled out obstructive coronary artery disease. Further assessment with cardiac magnetic resonance imaging demonstrated a persistent communication between the aortic isthmus and the main pulmonary artery, confirming PDA. A diagnosis of acquired dilated cardiomyopathy secondary to long-standing left-to-right shunt, likely precipitated by frequent atrial and ventricular arrhythmias, was established. Although the patient was a candidate for transcatheter percutaneous ductal closure, he declined any intervention, and optimized guideline-directed medical therapy was initiated. This case represents an exceptionally rare presentation of PDA in adulthood and highlights the importance of lifelong medical surveillance in patients with congenital heart defects.

## Introduction

The ductus arteriosus (DA), also referred to as ductus Botalli after the Italian physician Leonardo Botallo, is a transient, 5-10 mm long, vascular structure, vital for fetal life [1,2]. It connects the trunk of the pulmonary artery with the proximal descending aorta, at the level of the left subclavian artery, allowing blood to bypass the nonfunctioning lungs [3]. It develops early during embryogenesis, between the sixth and eighth week of gestation, and remains essential throughout intrauterine circulation by ensuring adequate systemic perfusion.

In utero, oxygenated blood from the placenta reaches the right atrium through the inferior vena cava. A substantial portion crosses the foramen ovale into the left atrium. The remaining blood, together with venous return from the superior vena cava, passes into the right ventricle and is subsequently propelled into the pulmonary artery. Owing to high pulmonary vascular resistance, it is largely diverted through the DA into the systemic circulation. Only a small fraction, approximately 10%, is ejected to the pulmonary circulation [4]. This arrangement permits efficient oxygen delivery to vital organs while limiting pulmonary blood flow.

Maintenance of ductal patency during fetal life depends on several interacting factors [3,5]. Low arterial oxygen tension, increased resistance of the pulmonary vascular bed, and locally acting vasodilators prevent smooth muscle constriction. Prostaglandins, particularly prostaglandin E2 (PGE2) and to a lesser degree prostaglandin I2 (PGI2), also known as prostacyclin, play a dominant role by promoting relaxation of the ductal wall through specific receptors. Additional mediators include nitric oxide, adenosine, atrial natriuretic peptide, and carbon dioxide. At birth, major circulatory changes induce ductal closure [5]. Lung expansion by inhalation reduces pulmonary vascular resistance, while removal of the placenta increases systemic resistance. Arterial oxygen levels rise, and placental prostaglandin supply ceases, while remaining PGE2 is rapidly metabolized in the lungs. These combined effects result in functional closure within 12 to 24 hours, followed by anatomical obliteration over the subsequent two to three weeks. Eventually, DA becomes a fibrous structure, the ligamentum arteriosus [3].

Failure of closure results in patent ductus arteriosus (PDA), a congenital cardiac abnormality that affects approximately one in 2,000 full-term live births and is observed in 20%-60% of preterm infants [6]. After birth, the reversal of pressure gradients promotes blood flow from the systemic into the pulmonary circulation, resulting in a left-to-right shunt [5]. Left untreated, and depending on its magnitude, it leads to complications. In the short term, it can cause hypoperfusion of vital organs, congestive heart failure, pulmonary edema and pulmonary hemorrhage due to volume overload, or even death [3,5,7]. Chronic shunting can be a site of infective endocarditis and a cause of pulmonary hypertension and heart failure [7]. Long-standing right ventricular pressure overload promotes hypertrophy [8]. Similarly, persistent left ventricular volume overload promotes its eccentric hypertrophy [7,9]. Although compensatory initially, these changes increase myocardial oxygen demand and, ultimately, systolic function is impaired. In extreme cases, acquired dilated cardiomyopathy may develop.

Although PDA is well studied in neonates and children, its persistence into adulthood is rare. In these uncommon cases, even moderate shunts can gradually overload the heart, causing the ventricles to adapt structurally. Over time, this chronic strain may weaken the myocardium, eventually leading to acquired dilated cardiomyopathy [7]. Highlighting such cases is important for recognizing potential late complications in adults. Due to routine neonatal screening with physical examination and echocardiography and early intervention when indicated, persistence of PDA into adulthood is uncommon [10]. Therefore, the above long-term complications have been largely prevented. Nevertheless, isolated cases continue to appear. Here, we describe a rare presentation of acquired dilated cardiomyopathy in a 70-year-old male resulting from a moderate left-to-right shunt due to PDA.

## Case presentation

A 70-year-old male was admitted to the cardiology emergency department with progressive dyspnea, dry cough, and bilateral lower limb swelling, worsening for one month. He also reported orthopnea for the last two days, which prompted him to seek medical attention. He denied chest pain, fever, symptoms suggestive of recent infection, or other complaints. The patient was an active smoker with 50 pack-years. His medical history was notable for PDA diagnosed in childhood, for which he denied any intervention, and subsequently, he reported the absence of regular medical follow-up. His family history was unremarkable for cardiovascular disease or cardiomyopathies.

**Figure 1:**
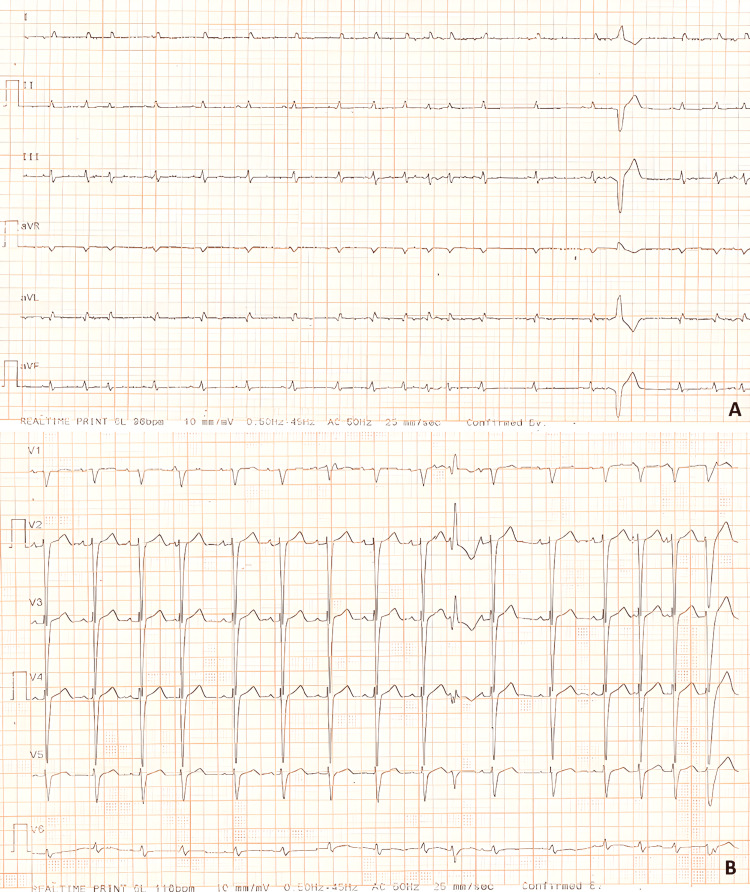
A 12-lead electrocardiogram of the patient demonstrating sinus rhythm at approximately 110 beats/minute with premature atrial and ventricular beats. In panel A, nonspecific ST-T segment changes are present, whereas panel B shows poor R-wave progression.

At the time of evaluation, the patient was afebrile but exhibited tachypnea, with 25 breaths/minute. Blood pressure was measured at 135/70 mmHg, and his heart rate was approximately 110 beats/minute. Electrocardiography revealed a sinus rhythm with frequent atrial and ventricular premature beats (Figure 1). There was poor R-wave progression in the precordial leads and nonspecific ST-T segment abnormalities in the limb leads, without features suggestive of a specific cardiac disorder. Arterial blood gas analysis obtained without supplemental oxygen is shown in Table 1. The analysis showed hypocapnia, metabolic acidosis, and increased lactate, reflecting a compensated metabolic acidosis due to tissue hypoxia, with respiratory compensation through tachypnea. Physical examination revealed diaphoresis, orthopnea, jugular venous distention, and marked bilateral lower limb edema up to the knees. The apical impulse was laterally displaced. Cardiac auscultation demonstrated a prominent S2 component, an S3 sound, and mild systolic murmurs at the site of the tricuspid and mitral valves, suggestive of regurgitation. The patient also had a continuous “machinery” murmur of grade 3/6, best heard at the left subclavicular area, which radiated to the back, suggestive of PDA. Lung auscultation showed bilaterally reduced breath sounds, absent at the bases, with wet crackles over the mid-pulmonary fields.

**Table 1:** Arterial blood gas analysis results.

Parameter	Measurement unit	Value	Normal range
pH	-	7.37	7.35–7.45
Carbon dioxide partial pressure (pCO_2_)	mmHg	26.3	35–45
Oxygen partial pressure (pO_2_)	mmHg	91.7	80–100
Hemoglobin saturation (SO_2_ %)	%	97.1	95–100
Bicarbonate (HCO_3_^-^)	mmol/L	15.5	22–26
Lactate	mmol/L	3.8	0.5–2.2

**Figure 2:**
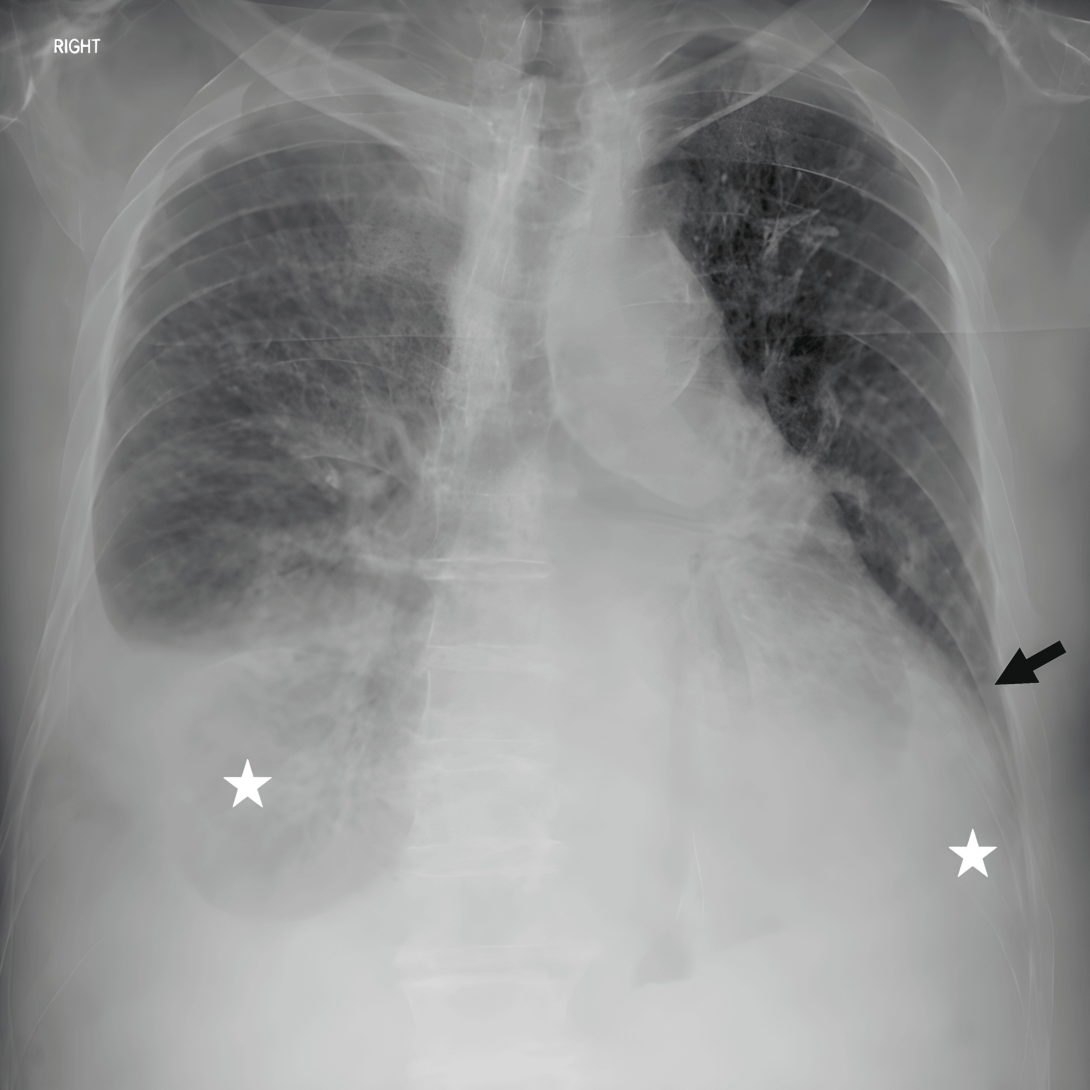
Chest radiograph of the patient in the upright position demonstrating cardiomegaly (black arrow) and pleural effusions on both sides (white asterisks), predominantly on the right.

Chest radiography demonstrated cardiomegaly, pulmonary vascular cephalization, alveolar edema, and pleural effusions on both sides, with greater accumulation on the right (Figure 2). Laboratory analysis is shown in Table 2. It indicated a normal white blood cell count of 5,810/μL with 88% neutrophils, as well as normal hemoglobin, hematocrit, and platelet count. Renal function was mildly impaired with a creatinine level of 1.4 mg/dL. High-sensitivity troponin I was mildly elevated, and brain natriuretic peptide was also increased. Electrolytes, liver biochemistry, inflammatory markers, lipid profile, and thyroid function tests were within normal limits.

**Table 2:** Laboratory findings.

Parameter (measurement unit)	Value	Normal range
White blood cells (/μL)	5,810	4,700–10,500
Neutrophils (%)	88	40–75
Lymphocytes (%)	6.7	20–40
Monocytes (%)	4.5	2–10
Eosinophils (%)	0.2	1–6
Basophils (%)	0.2	<2
Hemoglobin (g/dL)	15.1	13.5–17
Hematocrit (%)	43.5	40–51
Platelets (/μL)	179,000	140,000–450,000
International normalized ratio	1.1	-
D-dimers (mg/L)	0.2	<0.5
Total cholesterol (mg/dL)	185	140–240
Low-density lipoprotein (mg/dL)	121	60–130
High-density lipoprotein (mg/dL)	35	35–65
Triglycerides (mg/dL)	85	45–170
Thyroid-stimulating hormone (μUI/mL)	2.6	0.5–5
Brain natriuretic peptide (pg/mL)	1,220	0.1–100
Glucose (mg/dL)	113	75–150
Urea (mg/dL)	71	15–50
Creatinine (mg/dL)	1.4	0.6–1.3
Uric acid (mg/dL)	6.7	3.6–7.2
Potassium (mEq/L)	4.1	3.5–5.5
Sodium (mEq/L)	138	135–150
Calcium (mEq/L)	8.6	8.5–10.6
Magnesium (mEq/L)	2.0	1.3–2.1
Troponin I (ng/L)	143	<54
Creatine phosphokinase (IU/L)	135	35–300
Aspartate aminotransferase (IU/L)	35	5–40
Alanine transaminase (IU/L)	34	5–45
Alkaline phosphatase (IU/L)	81	30–125
Gamma-glutamyl transferase (IU/L)	33	5–37
Total bilirubin (mg/dL)	0.6	0.4–1
Amylase (IU/L)	48	1–95
C-reactive protein (mg/dL)	0.4	<0.5

**Figure 3:**
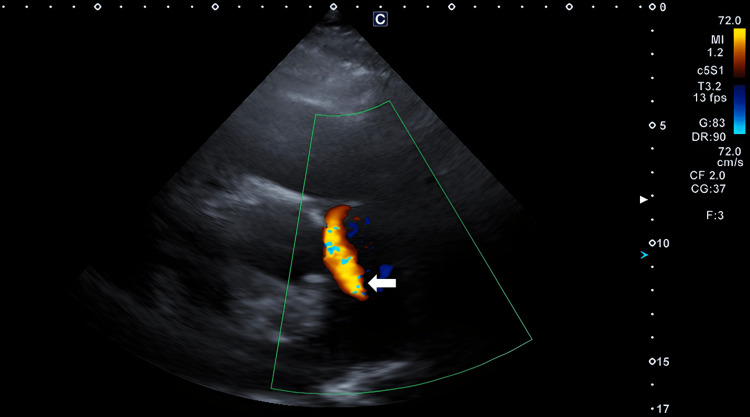
Transthoracic echocardiogram in the parasternal short-axis view demonstrating abnormal flow into the main pulmonary artery due to the presence of patent ductus arteriosus (white arrow).

**Figure 4:**
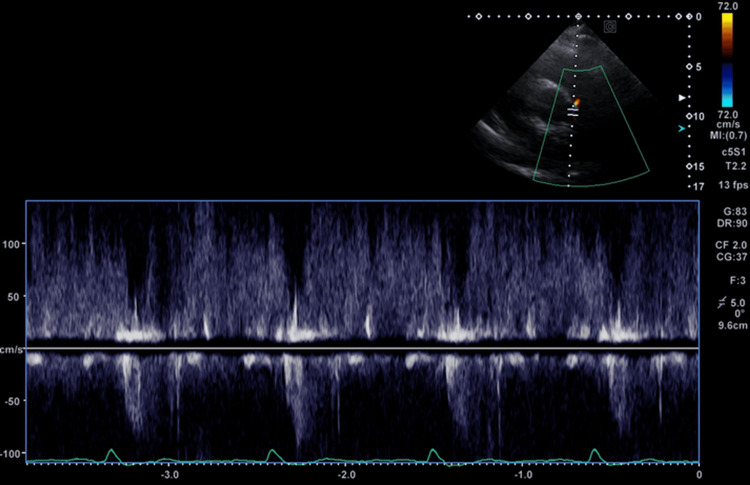
Pulse-wave Doppler demonstrating continuous reversed flow in the pulmonary artery, consistent with a left-to-right shunt.

Intravenous diuretic therapy was initiated promptly. After hemodynamic stabilization, transthoracic echocardiography was performed (Figures 3, 4). All four cardiac chambers were dilated, with severely compromised biventricular systolic performance. Left ventricular ejection fraction (LVEF) was estimated at 25%, with eccentric hypertrophy (intraventricular septum: 0.8 cm, posterior wall: 0.6 cm, left ventricular end-diastolic diameter: 6.2 cm). Right ventricular systolic velocity (RV S′) was 6.5 cm/s. Mild insufficiency of mitral and tricuspid valves was present, and the pulmonary artery systolic pressure (PASP) was 45 mmHg. Qp/Qs was calculated at 1.6, indicating a moderate left-to-right shunt. The patient was admitted to the cardiology ward, and medical therapy for heart failure with reduced ejection fraction (HFrEF) was started, following current guideline recommendations [11]. During hospitalization, the patient was monitored on telemetry, and episodes of paroxysmal atrial fibrillation were documented. Therefore, oral anticoagulation therapy with a new oral anticoagulant was added, after evaluating stroke risk according to the CHA₂DS₂-VASc score [11].

**Figure 5:**
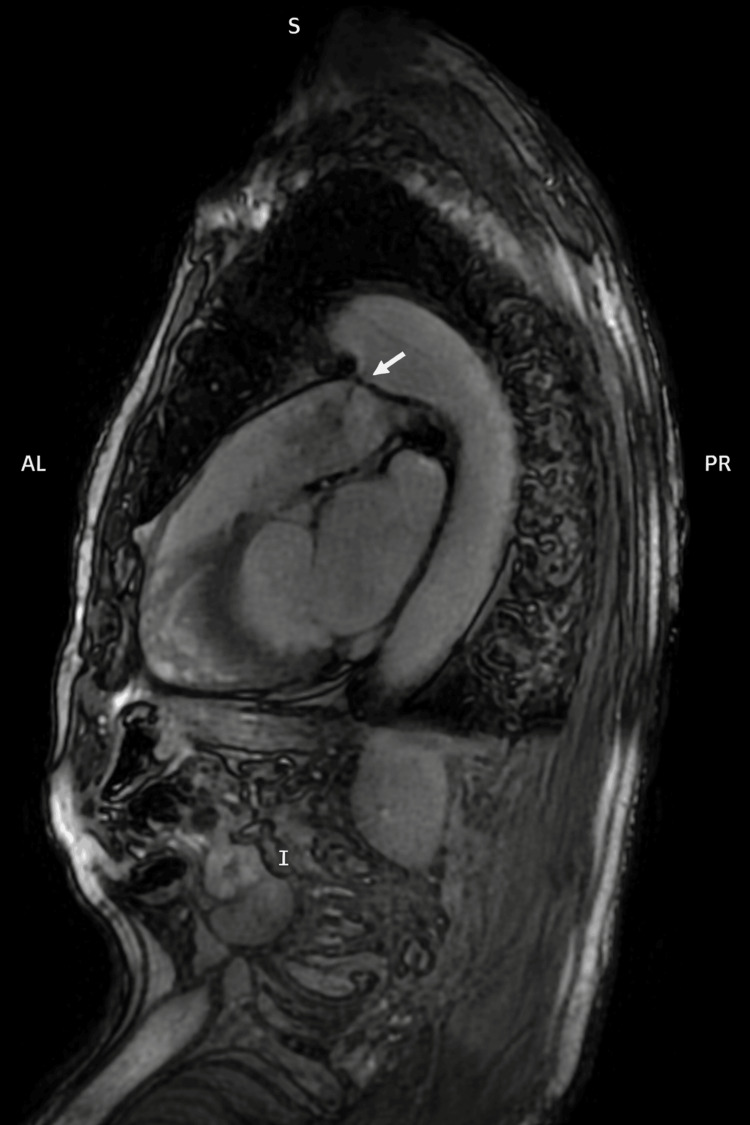
Cardiac magnetic resonance imaging of the patient in sagittal view showing a communication between the aortic isthmus and the main pulmonary artery (white arrow), confirming the diagnosis of patent ductus arteriosus.

Our differential diagnosis of the patient’s HFrEF included ischemic, infectious (such as prior myocarditis), toxic (such as excessive alcohol consumption), hypertensive, valvular, arrhythmia-induced, shunt-related, infiltrative, and genetic causes [11]. The patient’s history and initial evaluation allowed exclusion of most etiologies. To clarify the underlying cause of the patient’s pronounced systolic impairment, coronary angiography was conducted, which found no evidence of significant coronary obstruction, excluding an ischemic cause. Subsequently, cardiac magnetic resonance imaging (MRI) was performed for further evaluation. It confirmed the initial findings and excluded the presence of myocardial scarring, myocardial edema, findings suggestive of infiltrative disease, or other concomitant congenital heart disease (Figure 5). Therefore, we zeroed in on the diagnosis of an acquired dilated cardiomyopathy due to the chronic exposure to a moderate left-to-right shunt. We hypothesized that frequent premature atrial and ventricular extrasystoles, which we detected during telemetry, as well as possible episodes of high-rate paroxysmal atrial fibrillation, also contributed to the patient’s condition.

**Figure 6:**
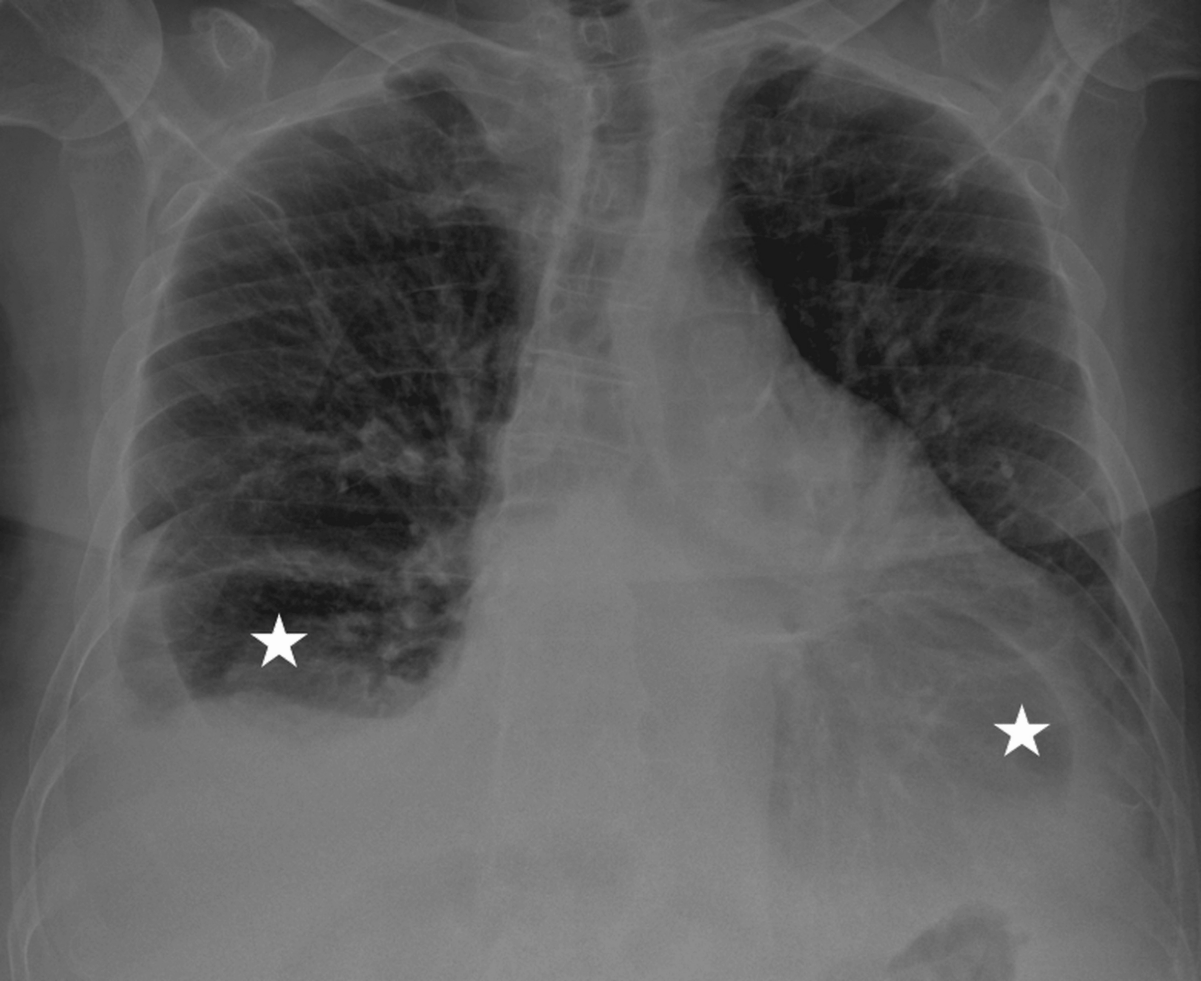
Chest radiograph obtained at the one-month follow-up showing marked improvement of the previously noted bilateral pleural effusions (white asterisks).

Despite our strong recommendations, the patient refused invasive treatment and chose conservative medical management. He was discharged on day 10 on diuretic therapy, guideline-directed medical therapy for HFrEF, rhythm control, and anticoagulation. At the one-month follow-up, he reported no dyspnea or other symptoms. A repeat chest radiography demonstrated significant resolution of pleural effusions (Figure 6). At three months, with good adherence to his medications, improvement in ventricular systolic function was noted, with LVEF increasing to 32%, and RV to S’ 7.3 cm/s. PASP was estimated at 40 mmHg.

## Discussion

PDA occurs in approximately 1 in 500 to 1 in 2,000 live births and is more frequently observed in premature neonates or in association with other forms of congenital heart disease [12]. Advances in early diagnosis and timely intervention have markedly reduced its persistence into adulthood and have significantly limited long-term complications. In the present case, no additional congenital abnormalities were identified, and a precise cause for persistent ductal patency, including prematurity, could not be established.

When left untreated, PDA may impose a substantial hemodynamic burden. Its impact on the adult cardiovascular system has been previously described and has been associated with complications such as infective endocarditis, with an estimated incidence of approximately 1% among patients with PDA, ductal aneurysm formation, and heart failure [7]. In adult patients, heart failure most commonly presents with preserved or only moderately reduced LVEF [12-16]. Cases with severely reduced LVEF are rarely reported. The most extreme cases described in the literature include a 92-year-old female with an LVEF of 39% and an 88-year-old female with an LVEF of 27% [17,18]. To our knowledge, an LVEF of 25% represents the lowest reported value of HFrEF in adulthood directly attributed to long-standing PDA. The moderate magnitude of the shunt could explain the impaired ventricular function in our patient. Frequent atrial and ventricular arrhythmias may also have contributed to the development of HFrEF.

The hemodynamic consequences of PDA are closely related to the magnitude of the shunt, which is determined by the size of the ductus and primarily by the relative flow through the pulmonary and systemic circuits (Qp/Qs) [5]. A lower ratio of pulmonary to systemic vascular resistance results in a larger left-to-right shunt. Larger ducts with lower resistance permit greater shunt volumes and more pronounced clinical consequences [7,19]. Small-sized or silent PDAs, with Qp/Qs < 1.5, usually do not cause significant hemodynamic compromise and may remain asymptomatic, often not requiring intervention. In contrast, moderate (ratios between 1.5 and 2.2) or large (ratios over 2.2) shunts may lead to pulmonary overcirculation, pulmonary hypertension, heart failure, and end-organ hypoperfusion.

In long-standing cases, sustained pulmonary overflow induces irreversible changes in the pulmonary vasculature, including medial hypertrophy of arterioles, intimal proliferation, and progressive fibrosis [7]. Ultimately, destruction of arterioles and capillaries occurs, leading to pulmonary vascular resistance that exceeds systemic resistance. At this stage, shunt flow becomes bidirectional and may eventually progress to Eisenmenger syndrome. In the bidirectional phase, blood flow through the ductus reverses during early systole from the pulmonary artery to the aorta and reverts during late systole and diastole. In advanced Eisenmenger physiology, a permanent right-to-left shunt is established throughout the cardiac cycle, resulting in systemic hypoxemia and cyanosis.

Patient presentation may include shortness of breath, fatigue with reduced exercise tolerance, ankle edema, palpitations, chest pain or discomfort, and recurrent respiratory infections [17]. Atrial fibrillation is also common in this population, a finding that was confirmed during hospitalization in our case. PDA can be suspected through careful physical examination and confirmed with appropriate imaging modalities. On clinical examination, patients with Eisenmenger syndrome secondary to PDA may demonstrate distinctive phenotypic characteristics compared with those with Eisenmenger physiology from other causes [20]. A characteristic finding is differential cyanosis, with lower oxygen saturation and clubbing of the toes and sparing of the fingers, reflecting the anatomical position of the ductus distal to the origin of the subclavian arteries. These features were not observed in our patient, as there was no evidence of Eisenmenger syndrome. The Qp/Qs ratio was estimated at 1.6, indicating a moderate left-to-right shunt capable of imposing a hemodynamic burden. If left untreated, this may progressively worsen pulmonary hypertension.

Cardiac auscultation plays a key role in the initial assessment. A persistent “machinery” murmur, located at the left upper sternal border and radiating to the back, is considered the hallmark of PDA [12]. The murmur typically intensifies during systole and attenuates but remains audible during diastole. Its intensity and configuration, particularly the relative systolic and diastolic components, reflect the hemodynamic significance of the shunt and its impact on the pulmonary circulation. In early stages, particularly during infancy, the diastolic component may be absent. As systemic vascular resistance exceeds pulmonary resistance throughout the cardiac cycle, the classic continuous murmur develops, often accompanied by a wide pulse pressure and, in severe cases, a palpable thrill. With the progression of pulmonary hypertension, the diastolic component diminishes and eventually disappears as bidirectional shunting occurs. Concurrently, the systolic component becomes less prominent and the second heart sound (S2) intensifies. In Eisenmenger syndrome, the murmur may be entirely absent.

Electrocardiographic findings in adults with PDA are generally nonspecific, ranging from a normal electrocardiogram in small shunts to evidence of left ventricular or biventricular hypertrophy and left atrial enlargement in more advanced cases [12]. Chest radiography may be unremarkable or may demonstrate cardiomegaly and radiographic signs of pulmonary hypertension, such as enlargement of the pulmonary arteries [2]. Signs of pulmonary congestion, as observed in our case, may be present in patients presenting with acute heart failure.

Echocardiography remains a cornerstone in the diagnosis and evaluation of PDA [7]. In infants and children, transthoracic echocardiography is usually sufficient to assess ductal anatomy and hemodynamic impact. In adults, however, acoustic window limitations may reduce diagnostic accuracy, and transesophageal echocardiography, particularly upper esophageal views, may be required. Routine assessment does not typically necessitate advanced techniques such as cardiac computed tomography or MRI, but provides valuable additional information in selected cases, including assessment of ductal calcification, aneurysm formation, or pre-procedural planning [2]. These modalities are also particularly useful in cases with systolic right-to-left shunting, where color Doppler imaging may be misleading due to adjacent flows [7].

The prognosis of patients with PDA largely depends on the magnitude of the shunt [12]. However, in all cases, there remains a risk of developing infective endocarditis. Silent shunts typically do not result in hemodynamic compromise during childhood or adulthood and remain asymptomatic. Moderate shunts may remain clinically silent during childhood but can manifest later in life with heart failure or pulmonary hypertension, most commonly during the third decade. Larger shunts usually become symptomatic in infancy.

Currently, transcatheter percutaneous closure devices, such as the Amplatzer and Nit-Occlud devices, represent the treatment of choice for PDA in symptomatic infants, children, and adults [2,12]. Asymptomatic patients with significant left heart volume overload are also considered candidates for closure. According to European guidelines, closure is recommended in patients with a left-to-right shunt, provided that pulmonary resistances are ≤5 Wood units and the Qp/Qs ratio is >1.5 [7]. In addition, coil devices may be used for the closure of small-sized PDAs. Surgical ligation or division is reserved for very large ducts or for premature neonates in whom pharmacological closure has failed [5]. Patients with markedly increased pulmonary vascular resistance and advanced pulmonary hypertension, such as those with Eisenmenger syndrome, are not candidates for closure and may instead receive palliative medical therapy, including advanced pulmonary vasodilator agents such as antagonists of endothelin receptors and inhibitors of phosphodiesterase-5. Here, although our patient was eligible for transcatheter percutaneous intervention, his preference for conservative management led us to initiate optimized medical therapy with close follow-up.

## Conclusions

Although a left-to-right shunt due to PDA is a well-recognized cause of heart failure, its detection in adulthood is rare. Only sporadic cases have been reported in the literature, most of which describe patients with preserved or moderately compromised LVEF. To our knowledge, acquired dilated cardiomyopathy with severely reduced LVEF secondary to long-standing PDA has been reported only rarely. Even though other contributing factors, such as frequent arrhythmias, could not be entirely excluded, this case represents an exceptionally rare presentation. This case highlights the importance of long-term medical surveillance in patients with congenital heart defects such as PDA, even when symptoms are absent early in life. It also emphasizes the need for timely and appropriate management to prevent progressive hemodynamic deterioration and irreversible myocardial damage. Furthermore, it underscores that late presentation of congenital heart disease should remain part of the differential diagnosis in adult patients presenting with unexplained heart failure.
